# ﻿*Ardisiakrauensis*, a new species of Primulaceae (Myrsinoideae) from Peninsular Malaysia

**DOI:** 10.3897/phytokeys.234.106829

**Published:** 2023-10-23

**Authors:** Avelinah Julius, Suhaimi Syahida-Emiza, Timothy M. A. Utteridge

**Affiliations:** 1 Institute for Tropical Biology and Conservation, Universiti Malaysia Sabah, Jalan UMS, 88400 Kota Kinabalu, Sabah, Malaysia Universiti Malaysia Sabah Kota Kinabalu Malaysia; 2 Forest Research Institute Malaysia, Kepong, Selangor, 52109, Malaysia Forest Research Institute Malaysia Kepong Malaysia; 3 Royal Botanic Gardens, Kew, Richmond, TW9 3AE, UK Royal Botanic Gardens London United Kingdom

**Keywords:** *Ardisiarigida*, conservation, endemic, Krau Wildlife Reserve, Pahang, SE Asia, subgenus *Pyrgus*, taxonomy

## Abstract

*Ardisiakrauensis*, a new species of Primulaceae from Peninsular Malaysia, is described and illustrated. The new species is assignable into subgenus (§) *Pyrgus* on account of specialised lateral reproductive branches bearing a terminal inflorescence subtended by foliose bracts. Morphologically, the new species mostly resembles *Ardisiarigida* in having elliptic leaves. However, the new species can be distinguished by the combination of its lateral veins number, the inflorescence branching pattern, the rachis and flower colour, and the stigma shape. *Ardisiakrauensis* is found in an entirely protected habitat, thus, it is assessed as Least Concern (LC).

## ﻿Introduction

*Ardisia* Sw., comprising about 730 species (POWO 2023) is one of the largest genera in the subfamily Myrsinoideae of the enlarged Primulaceae (APG 2016). The genus is morphologically classified into 14 subgenera by Mez (1902), with three additional proposed subgenera by Stone (1993: §*Scherantha* B.C.Stone, endemic to the Philippines), Larsen and Hu (1995: §*Tetrardisia* (Mez) K.Larsen & C.M.Hu) and Yang and Hu (2022: §*Odontophylla* (Y.P.Yang) C.J.Yang & J.M.Hu) using characters of habit, leaf morphology, disposition of flowers at inflorescence branch apices (racemes, umbels, corymbs), inflorescence position and floral morphology. Of these, eleven subgenera are present in Peninsular Malaysia (for the grouping discussion see Stone 1989a; Larsen and Hu 1995; Yang and Hu 2022).

A flowering plant of *Ardisia* was collected during a botanical survey led by the second author in Krau Wildlife Reserve, Pahang in 2022. The plant is assignable to §*Pyrgus* (Lour.) Mez, which is defined by the combination of the small, woody shrub habit (rarely trees), entire leaves lacking bacterial nodules, and specialised lateral reproductive branches bearing a terminal inflorescence subtended by leaf-like foliose bracts (or referred to as ‘reproductive shoot’) (see Julius et al. 2021a; Utteridge and Julius 2022). This subgenus only has two species in Peninsular Malaysia: *Ardisiacalophylla* Furtado and *A.rigida* Kurz s.l.; the latter is widespread from Peninsular Malaysia, through the Andamans (the type locality of *A.rigida*) and into the western side of Thailand. In the last treatment of the genus in Peninsular Malaysia, Stone (1989a) listed *A.calophylla* and *A.vaughanii* Ridl. as the two members of §*Pyrgus*. However, comparison of the types of *Ardisiavaughanii* and *A.rigida* shows clearly that they are conspecific, with *A.rigida* the older name, and both taxa were sampled for a recent molecular study and were found to be sister taxa with little or no support to maintain them as distinct species (Julius et al. 2021b). In addition, *A.oxystemon* Ridl. ex H.R.Fletcher was described from Peninsular Thailand but is conspecific with *A.rigida* and was placed as a synonym of the latter in the recent Flora of Thailand treatment (Larsen and Hu 1996).

After morphological comparison to closely related species and consultation with relevant literature (Furtado 1959; Stone 1989a, 1989b; Larsen and Hu 1996), the new taxon is an undescribed species and is thus described and illustrated here as new to science.

## ﻿Material and methods

Morphological comparison with related species, viz. *Ardisiacalophylla* and *A.rigida* s.l. (including *A.oxystemon* and *A.vaughanii*), was based on the study of herbarium material at K and KEP (acronyms according to Thiers 2016). In addition, specimen images online were also consulted (http://plants.jstor.org/). Floral measurements were made from rehydrated specimens. Morphological description of the new species is following Utteridge and Julius (2022). Flowering and fruiting material is indicated by ‘fl.’ and “fr.”, respectively. The conservation status of the new species was assessed following IUCN standards (IUCN 2012, 2022), including guidelines and procedures developed by FRIM for the Malaysia Plant Red List (Chua and Saw 2006).

## ﻿Taxonomy

### ﻿ Ardisia krauensis

Taxon classificationPlantaeEricalesPrimulaceae

Julius, Syahida-Emiza & Utteridge, sp. nov. (§ Pyrgus)

ABAA8C3E-6BEF-5F44-A6D6-A7F75B8BA5F4

urn:lsid:ipni.org:names:77329057-1

Figs 1 , 2

#### Diagnosis.

Similar to *Ardisiarigida* in having elliptic leaves but differs in the lateral veins arranged more or less in parallel (vs. ascending), the specialised lateral branch with only 1 or 2–3 foliose bracts along its length (vs. 2–4), its inflorescence branched to 3-ordered (vs. branched to 2-ordered), pendulous with the rachis green and thinner (vs. erect, pink and stout), corolla lobes spreading (vs. recurved) and the stigma trilobed (vs. punctiform).

**Figure 1.**
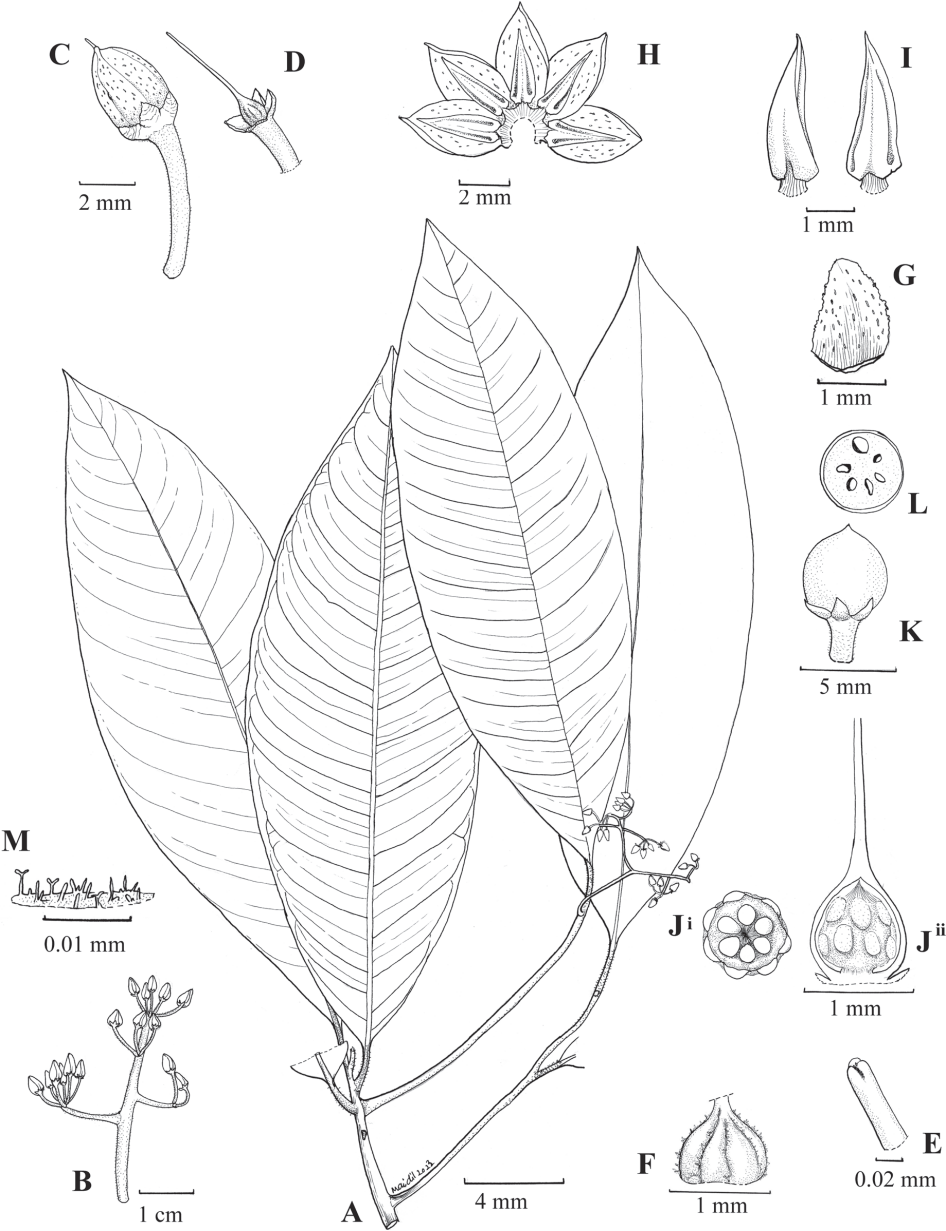
*Ardisiakrauensis***A** flowering branches **B** inflorescence **C** flower bud **D** corolla removed showing pistil **E** close-up of stigma **F** the grooved and hairy young ovary **G** abaxial view of calyx lobe **H** flower (spread) showing the stamen arrangement **I** abaxial (left) and adaxial (right) view of anther **J** dorsal (left) and ventral (right) view of placenta with ovules **K** young fruit **L** cross-section of young fruit **M** hair details. Illustration by Mohd Aidil Nordin.

#### Type.

**Figure 2.**
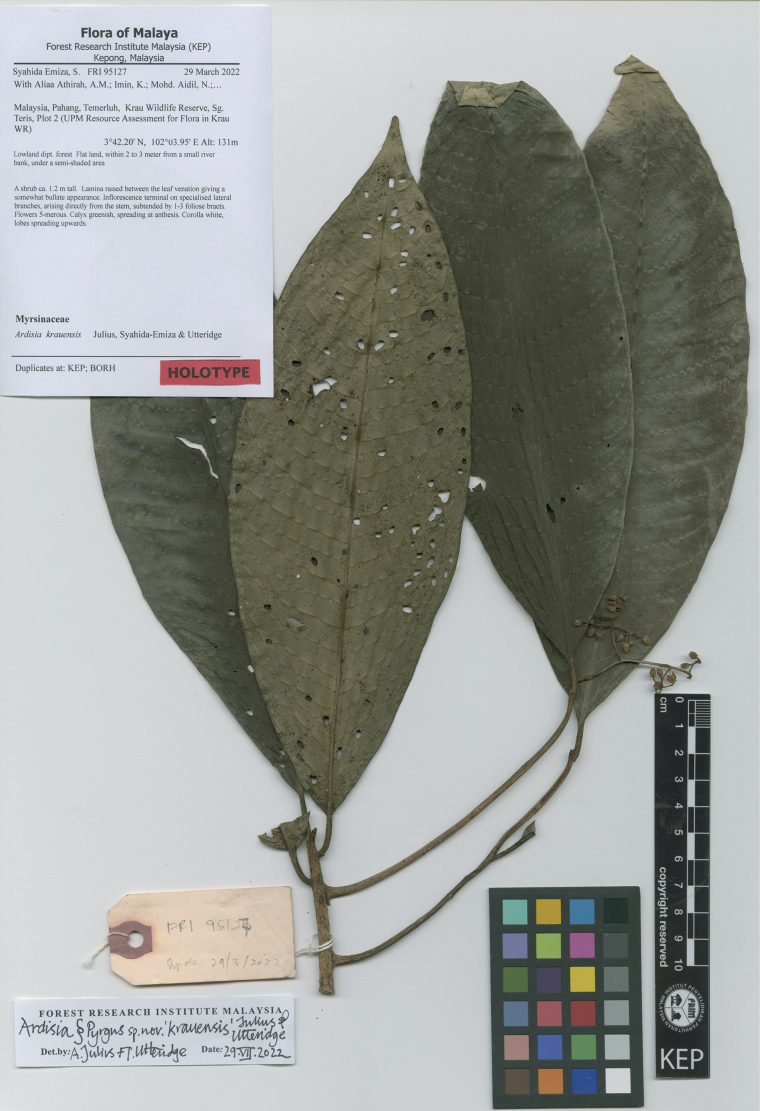
The holotype image of *Ardisiakrauensis*.

Malaysia. Peninsular Malaysia: Pahang, Temerluh, Krau Wildlife Reserve, Sg. Teris, Plot 2 (UPM Resource Assessment for Flora in Krau Wildlife Reserve), 3°42.20'N, 102°03.95'E, alt. 131 m, 29 March 2022 (fl. & young fr.), *Syahida-Emiza et al. FRI 95127* (holotype KEP!; isotype BORH!) (Fig. 2).

#### Description.

Shrub *c.* 1.2 m tall. ***Indumentum*** of sessile, circular and rusty scale on vegetative part and multi-cellular ginger-brown hairs with two clavate lobes, and simple hairs of various lengths, either arranged singly or in groups of 2 or 3 on petiole and reproductive part. ***Leaves*** pseudowhorl of 3; petioles 1.5–1.7 cm long, covered with dense, rusty, simple and forked glandular hairs when young, glabrous when mature; lamina chartaceous, raised between venation giving a somewhat bullate appearance, elliptic, 18.5–26 × 5–9 cm, base cuneate, margin entire, apex acuminate, acumen 1–1.5 cm long, glabrous on both surfaces, but densely scaly beneath; midrib flat above, raised beneath; lateral veins 15–21 pairs, joining towards the margin, and 1–2 intersecondary veins, prominent on both surfaces; intercostal veins obscure above, faintly reticulate beneath. ***Inflorescences*** terminal on specialised lateral branches arising directly from the stem, with 1–3 foliose bracts along the length of the branch and only one subtending the inflorescence, bracts elliptic, 16–26 × 5.5–9 cm, base cuneate, apex acuminate with acumen *c.* 1 cm long, petiole 7–10 mm long, densely hairy when young, glabrous when mature; inflorescences pendulous, branched to 3-orders, 5–6.2 cm long; peduncle and rachis green, thin, covered with dense, rusty, simple and forked glandular hairs throughout. ***Flowers*** 5-merous; pedicels 4–7 mm long, densely hairy and scaly towards calyx; calyx-lobes, ovate, *c.*1.5 × 1 mm, margin erose, apex obtuse, spreading at anthesis, glabrous adaxially, densely hairy abaxially; corolla tube *c.* 0.2 mm long, lobes spreading, ovate, 3.8 × 2–3 mm, margin entire, one side slightly curve inward, apex appears acuminate but obtuse when flatten, glabrous on both surfaces but densely gland dotted throughout abaxially; stamens 5, filaments short *c.* 0.5 mm long at the basal part of anther, anthers ovate, *c.* 3 × 1.5 mm, connective acute, thecae not locellate, dehiscent by longitudinal slits; ovary subglobose, 0.5 × 0.8 mm, grooved at young stage, hairy on the groove, becoming smooth and glabrous with age, ovules *c.* 13 arranged in 2-series, stigma and style slender, 3.6–3.8 mm long, stigma trilobed, hairy with simple multicellular hairs adaxially, glabrous abaxially. ***Young fruit*** green, globose, *c.* 5 × 4.8 mm, glabrous, mature fruit unseen.

#### Distribution.

**Figure 3.**
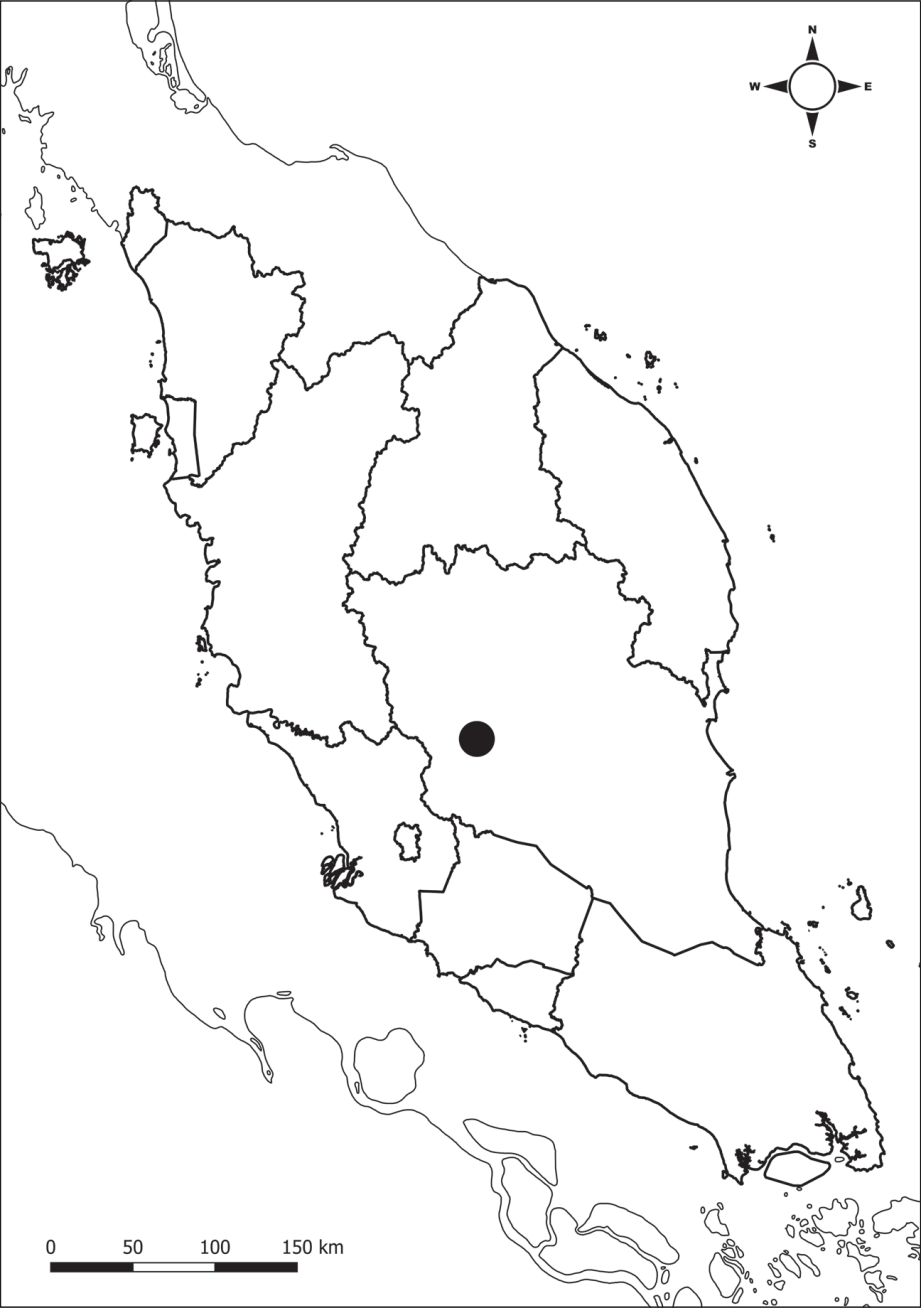
The type locality of *Ardisiakrauensis* in Pahang, Peninsular Malaysia (Black Dot).

Endemic to Peninsular Malaysia, Pahang. Thus far known only from Krau Wildlife Reserve (Fig. 3).

#### Ecology.

In lowland forest, flat land near small river under a semi-shaded area, at 131 m altitude. Flowers and young fruits in March.

#### Etymology.

The species epithet ‘krauensis’ refers to the type locality Krau Wildlife Reserve where it was collected.

#### Conservation status.

Least Concern (LC). This new species was collected during a general survey in Krau Wildlife Reserve in 2022. There is no survey conducted specifically for this species yet, therefore we could not estimate the number of mature individuals and assessed it against Criterion D. As for D2, although this species is only known from one locality, but the habitat is protected and no plausible threat is identified, so it does not meet D2 also. Therefore, this species is currently best assessed as of Least Concern.

#### Notes.

ArdisiasubgenusPyrgus is one of the smallest groups comprising only two known species in Peninsular Malaysia. This latest addition brings the number of §*Pyrgus* species native to Peninsular Malaysia to three including *A.calophylla* and *A.rigida*. The new species resembles *A.calophylla* in that both have inflorescences branched to three orders, but the leaves of the former species are much smaller (to 10 cm long and 4 cm wide), obovate with an acute to rounded tip and a coriaceous lamina, whereas the leaves of *A.krauensis* are larger (18.5–26 cm long and 5–9 cm wide), elliptic with a long acuminate tip and a chartaceous lamina. Compared to *A.rigida*, the new species differs in the denser venation comprised of more lateral vein pairs (15–21 pairs vs. usually less than 15 pairs in *A.rigida*, though occasionally up to 18 pairs) as well as 1–2 intersecondary veins. The leaves of *A.rigida* usually dry and sandy-brown with the venation somewhat obscure, giving a very ‘flat’ and dull appearance in the herbarium, whereas the leaves of *A.krauensis* are olive green when dry, are slightly thinner and dry with a ‘puckered’ appearance along the conspicuous venation. In *A.rigida*, the specialised lateral branches have up to 4 foliose bracts along their length and the terminal inflorescence is stout with a rigid rachis to 15 cm long; this is very different from the lateral branches with 1–3 foliose bracts and a thinner and more pendulous inflorescence rachis to only 5–6.2 cm long. The flowers of the new species differ with the filaments that are positioned at the base of the anther (vs. peltate), and the anther connective acute (vs. connective elongated, narrowly acute and becoming recurved at anthesis).

The stigma of *Ardisia* has variously been described as punctiform (Stone 1989a) or apiculate (Utteridge 2021), but *A.krauensis* possesses a stigma that is distinct in that it is divided into three lobes, closed, and hairy with simple multicellular hairs adaxially but glabrous abaxially. For any species within the genus that we previously studied, the ovary often exhibits a constant shape and degree of hairiness. The ovary of *A.krauensis*, however, is grooved and hairy at the groove when young (Fig. 1F), but gradually becomes smooth and glabrous with age (Fig. 1J^ii^).

### ﻿Key to Peninsular Malaysian species of Ardisiasubgen.Pyrgus

**Table:** 

1	Leaves up to 10 cm long, lamina obovate with acute to rounded apex, coriaceous; foliose bracts in a pseudowhorl of 3–5 just below the inflorescence	***Ardisiacalophylla***
–	Leaves > 10 cm long, lamina elliptic with obtuse or acuminate apex, chartaceous; foliose bracts alternately arranged 1–4	**2**
2	Foliose bracts 2–4; inflorescences erect, branched to 2-ordered; rachis pink and stout; corolla lobes recurved; stigma punctiform	***Ardisiarigida***
–	Foliose bracts 1–3; inflorescences pendulous, branched to 3-ordered; rachis green and thinner; corolla lobe spreading; stigma trilobed	***Ardisiakrauensis***

## Supplementary Material

XML Treatment for Ardisia krauensis

## ﻿Additional information

### Author ORCIDs

Avelinah Julius https://orcid.org/0000-0003-1991-1915

Suhaimi Syahida-Emiza https://orcid.org/0000-0002-9321-2779

Timothy M.A. Utteridge https://orcid.org/0000-0003-2823-0337

### Data availability

All of the data that support the findings of this study are available in the main text.
